# Analysis of young students' perception of loneliness: a cross-sectional study

**DOI:** 10.3389/fpsyg.2025.1553490

**Published:** 2025-06-24

**Authors:** Rogelio Hernández-Díaz, Lucas Navarro-Vásquez, Alejandra Aguilar-Latorre, Fátima Méndez-López, Santiago Gascón-Santos, Rosa Magallón-Botaya

**Affiliations:** ^1^Department of Medicine, Psychiatry and Dermatology, Faculty of Medicine, University of Zaragoza, Zaragoza, Spain; ^2^Torrero Primary Care Health Center - Zaragoza II Sector, Aragonese Health Service, Zaragoza, Spain; ^3^Department of Psychology and Sociology, Faculty of Human Sciences and Education, University of Zaragoza, Huesca, Spain; ^4^Aragonese Primary Care Research Group (GAIAP), Institute for Health Research Aragón (IIS Aragón), Zaragoza, Spain; ^5^Research Network on Chronicity, Primary Care and Health Promotion RD24/0005/0004, RICAPPS, Madrid, Spain; ^6^Department of Physiatry and Nursing, Faculty of Health Sciences, University of Zaragoza, Zaragoza, Spain; ^7^Department of Psychology and Sociology, Faculty of Social and Human Sciences, University of Zaragoza, Teruel, Spain

**Keywords:** teenagers, young adults, loneliness, health, mental health

## Abstract

**Introduction:**

Loneliness, a multidimensional emotional experience resulting from unmet social needs, affects individuals across demographics and is particularly prevalent among youth. It can be social or emotional and is linked to developmental transitions, reduced social networks, mental health conditions, and excessive social media use. Unlike desired solitude, loneliness is involuntary and associated with significant physical and mental health risks, including depression, suicide, and chronic illnesses. Despite its public health impact, youth loneliness remains underrecognized, necessitating tailored interventions. This study examines its prevalence and relationship with sociodemographic factors, social support, social media use, self-esteem, and health among students in Aragon.

**Methods:**

This cross-sectional study investigated loneliness among adolescents and young adults (14–30 years) studying in Zaragoza, Spain, using online surveys conducted in March–April 2024. A sample of 536 participants was selected based on inclusion criteria, including informed consent. Loneliness was assessed using the UCLA Loneliness Scale and the De Jong Gierveld Loneliness Scale, alongside sociodemographic, social, and psychological variables such as self-esteem, health, mental health, and social media use. Descriptive, correlational, and regression analyses were performed to identify predictors of loneliness.

**Results:**

The study sample comprised 73.7% women, with an average age of 20 years. Among participants, 45.9% were high school students and 54.1% university students. Higher loneliness levels are associated with spending more time on social media, fewer and lower-quality relationships, lower self-esteem, poorer self-perceived health, and having mental health problems. While no significant gender or age differences were found, the UCLA Loneliness Scale identified 31.2% of participants as lonely, and the De Jong Gierveld Loneliness Scale classified 49.1% with moderate loneliness and 27.1% with severe loneliness.

**Discussion:**

This study highlights the high prevalence of loneliness among young individuals, affecting approximately two-thirds of the population aged 14–30. The findings underscore the importance of addressing loneliness as a public health concern, with particular attention to vulnerable groups. Further research is needed to develop effective prevention, detection, and intervention strategies tailored to youth, which could be implemented through Primary Care and educational institutions.

## Introduction

Loneliness is a complex and multifaceted emotional experience that emerges from the discrepancy between the social relationships individuals desire and those they actually have (Xia and Li, 2018). Defined by Montero and Sánchez (Montero et al., 2001) as “a potentially stressful multidimensional psychological phenomenon,” loneliness can arise from perceived or actual deficiencies in affective, social, or physical domains. It affects individuals of all ages, genders, and socio-economic backgrounds and can fluctuate in intensity, appearing temporarily, intermittently, or becoming chronic (Cacioppo and Cacioppo, 2012; Sota-Velásquez et al., 2021). The experience of loneliness is not static and manifests differently across contexts. It is important to differentiate between social loneliness, characterized by an absence of group belonging (Szapu et al., 2022), and emotional loneliness, which reflects a perceived lack of close and meaningful relationships (Montero et al., 2001; Szapu et al., 2022). Additionally, the distinction between desired solitude and unwanted loneliness is critical. Desired solitude can be enriching and rejuvenating, whereas unwanted loneliness is involuntary and often linked to isolation, exclusion, and significant emotional suffering (Díez Nicolás and Morenos Páez, 2015; World Health Organization, 2023). Unwanted loneliness has been recognized as a growing global public health concern. Studies in Spain reveal that 25.5% of young individuals aged 16 to 29 reports experiencing unwanted loneliness, with women and those aged 22 to 27 disproportionately affected (Tuñón et al., 2023). The prevalence of loneliness follows a U-shaped distribution, peaking during adolescence and old age, while being less common at intermediate life stages (Catalonia Red Cross, 2022; Tuñón et al., 2023). Despite this, research efforts have historically focused on the elderly, often neglecting youth loneliness, which remains an underexplored and invisible issue (Dumont et al., 1990).

Youth loneliness stems from a confluence of social, psychological, and environmental factors. Adolescence and early adulthood are periods marked by significant developmental and social transitions, such as entering university or the workforce, which often disrupt existing support networks (Luhmann and Hawkley, 2016). The quality and quantity of friendships and family ties play pivotal roles, as do socioeconomic challenges, immigration without a support network, and physical or mental disabilities (McIntyre et al., 2018; Thomas et al., 2020). Chronic mental health conditions such as anxiety and depression exhibit a bidirectional relationship with loneliness, both exacerbating and resulting from it. Personality traits such as shyness, pessimism, and low self-esteem further predispose individuals to loneliness (Mann et al., 2022). University students are particularly vulnerable during their first academic year, as they face heightened academic pressures, relocation challenges, and the need to establish new social networks (McIntyre et al., 2018).

The digital era has reshaped the social landscape, particularly for Generation Z, often referred to as “digital natives” (Álvarez-Ramos et al., 2019). Social media platforms provide opportunities for connection but also pose risks of fostering superficial interactions and unrealistic social comparisons. Excessive use of social media has been linked to poorer mental health outcomes and diminished real-world social engagement (Pérez and Quiroga-Garza, 2019). A survey by Zaragoza University (Government of Aragon, 2023) found that 38% of students used social media to cope with discomfort, often intensifying feelings of loneliness by reducing the time spent nurturing meaningful relationships (Government of Aragon, 2023). While social media is not inherently detrimental, its overuse can lead to dependency, poorer academic performance, and social isolation. Healthy use of technology—balancing online and offline interactions—is essential to mitigate its adverse effects (Cacioppo and Cacioppo, 2012; Echeburúa and De Corral, 2010).

Loneliness significantly affects both physical and mental health through direct and indirect mechanisms. Directly, it is associated with increased cortisol levels, systemic inflammation, and weakened immune responses. Indirectly, it contributes to unhealthy lifestyle behaviors such as poor diet, physical inactivity, and substance use (Holt-Lunstad et al., 2015; Steptoe et al., 2013). The UK Loneliness Commission equates the health risks of loneliness to smoking 15 cigarettes a day, underscoring its profound public health implications (Jo Cox Commission, 2017). The mental health consequences of loneliness are equally severe, encompassing heightened risks of anxiety, depression, and suicidal ideation (Mann et al., 2022). Adolescents and young adults experiencing loneliness report higher rates of self-harm and suicide attempts (Pearce et al., 2021). Additionally, loneliness exacerbates sleep disturbances, leading to fragmented and poor-quality rest, which further diminishes daytime functioning (Matthews et al., 2017). Chronic loneliness has also been implicated in long-term health outcomes, including cardiovascular disease, cognitive decline, and increased mortality. Addressing loneliness at an early age may be associated with better long-term health outcomes, potentially mitigating the risk of conditions such as cardiovascular disease, cognitive decline, and increased mortality (Valtorta et al., 2016).

Despite its profound impact, youth loneliness remains underrecognized in healthcare systems. Primary care services often prioritize loneliness interventions for older adults, leaving younger populations underserved (Gené-Badia et al., 2016). Collaborative approaches involving mental health professionals, educators, and community organizations are critical to addressing youth loneliness. Tailored interventions can significantly improve mental health outcomes, reduce social isolation, and enhance overall wellbeing (Masi et al., 2011).

Loneliness in youth can be better understood within established theoretical frameworks. The Biosocial Model of Health emphasizes the dynamic interplay between biological predispositions, psychological processes, and social environments in shaping health outcomes (Cacioppo et al., 2015; Engel, 1977). This model provides a useful lens through which to examine how factors such as digital hyperconnectivity, family structures, and mental health conditions interact to influence loneliness. Additionally, the Social Baseline Theory (Beckes and Coan, 2011) suggests that humans are biologically adapted to rely on social proximity to conserve energy and regulate affect. A lack of reliable social support systems may therefore lead to increased emotional and physiological costs, making adolescents and young adults particularly vulnerable to the effects of loneliness. In this context, the present study focuses on the region of Zaragoza (Aragón), which presents a relevant case study due to its distinctive demographic and social characteristics. Zaragoza (Aragon) has experienced recent demographic shifts, including increased youth migration and growing diversity in educational settings.

This study hypothesizes that loneliness is a prevalent but under-researched issue among adolescents and young adults, potentially driven by increased use of mobile phones, lack of social support, the impact of the COVID-19 pandemic and mental health conditions like anxiety and depression. The objectives are to determine the prevalence of loneliness among students in Aragon and to examine its association with sociodemographic factors, social support, social media usage, self-esteem, health, and mental health.

## Materials and methods

### Design

This study is a descriptive observational cross-sectional investigation.

### Population and sample

The target population comprises adolescents and young adults aged 14–30 years who are currently studying in high school or university in Zaragoza, Northern Spain. According to annual statistical reports from the Ministries of Education and Universities, this population consists of 5,570,725 students (Ministerio de Educación y Formación Profesional, 2023; Ministerio de Universidades, 2023). Based on a confidence level of 95%, a margin of error of 5%, and an assumed loneliness prevalence of 25.5% (Tuñón et al., 2023), a sample size of 292 participants was required.

The selection criteria included young individuals aged 14–30 years, enrolled in the 2023/2024 academic year in Compulsory Secondary Education, Baccalaureate, Intermediate Vocational Training, University Degree, Master's, or Doctorate programs, who agreed to participate in the study, provided informed consent, and accepted the Privacy Policy of the University of Zaragoza and Google. Exclusion criteria encompassed any individuals who did not meet these inclusion requirements.

### Procedure

Data were collected during March and April 2024 using two online surveys created specifically for this study via Google Forms. One survey targeted high school students, and the other university students. The questionnaire ensured anonymity and included both closed-ended questions with predefined answer options and a few open-ended questions. The first page provided the informed consent form and privacy policies from Google and the University of Zaragoza. Participants had to accept these to proceed. The survey for university students offered an optional short-answer question for personal contributions and an option to receive the study's results via email.

For university students, the survey was disseminated via social networks. Additionally, faculty representatives and professors assisted in encouraging participation. For high school students, school principals and administration from several high schools situated in the autonomous region of Zaragoza (Spain) facilitated the study after reviewing the proposal. Tutors invited students to complete the questionnaire during school hours using their computers or mobile devices. The high schools were chosen by convenience sampling (Galloway, 2005) and the sampling technique was non-probabilistic snowball sampling (Johnson, 2005). It should be noted that the sampling strategy, which relied on voluntary participation and online dissemination, may have introduced self-selection bias. This is particularly relevant considering the overrepresentation of female participants (73.7%), which could influence the generalizability of the results and should be considered when interpreting findings.

### Study variables

Sociodemographic variables included age (measured in years), gender (female, male), place/province of origin (Spain, foreigner), having or not having a partner, belonging to the LGTBIQ+ community (yes, no, prefer not to say), current educational level (high school or university), living situation (alone, with family or company), and participation in associative, sporting, solidarity, union or other activities (yes, no).

Variables related to loneliness included:

- The UCLA Loneliness Scale (Sheffield Hallam University, 2022) consists of three items that measure relational connection, social integration, and self-perceived isolation, with response options: “almost never,” “sometimes,” and “frequently” (replacing “often” for clarity). Scores range from 3 to 9 points, classifying individuals as not lonely (3–5 points) or lonely (6–9 points). Its validated Spanish version for the general population has a Cronbach's alpha of 0.78 (Pedroso-Chaparro et al., 2022), and in this study, reliability was also acceptable (alpha = 0.74).
- The De Jong Gierveld Loneliness Scale (Sheffield Hallam University, 2022) includes six items assessing emotional loneliness (lack of intimate relationships) and social loneliness (limited social networks). Responses are “yes,” “more or less (sometimes),” and “no.” Emotional loneliness items are scored as follows: “yes” and “more or less” = 1 point, “no” = 0 points; for social loneliness items, “yes” = 0 points, and “more or less” and “no” = 1 point. Total scores range from 0 to 6, categorizing individuals as not lonely (0–1 points), moderately lonely (2–4 points), or severely lonely (5–6 points). Although the Spanish version of this scale was originally validated for adults aged 60 and over (Ayala et al., 2012), we chose to include it in this study to explore its potential applicability in younger populations. No prior studies, to our knowledge, have validated the DJG scale specifically in adolescents or young adults. The Spanish version validated for individuals aged 60+ has a Cronbach's alpha of 0.77 (Ayala et al., 2012), with this study showing an acceptable reliability of 0.74.

Additional variables assessed include the frequency and duration of perceived loneliness, its associated causes, seeking help, the age group with the highest loneliness prevalence, its relationship with the COVID-19 pandemic, the societal importance of the issue, and the perceived quantity and quality of family, friendship, and peer/classmate relationships. Some questions were adapted from the State Observatory of Unwanted Loneliness's youth loneliness study questionnaire (Tuñón et al., 2023).

Other variables:

- Mobile phone and social media use: Daily time spent (in hours) and opinions on the relationship between loneliness and excessive internet use.
- Self-esteem, which is assessed using a Likert scale ranging from 1 to 5, with responses to the statement “I have high self-esteem” (strongly agree, agree, neutral, disagree, strongly disagree).
- Perceived health, rated on a Likert scale from 1 to 5, with options: very good, good, fair, bad, or very bad.
- Mental health problems, with the following response options: “No,” indicating the absence of mental health issues; “Yes, diagnosed by a doctor,” for individuals who have received a formal diagnosis; “Yes, I think I have a problem and have sought help,” for those who believe they have a mental health problem and have already sought professional assistance; and “Yes, I think I have a problem but have not sought help,” for individuals who acknowledge a mental health issue but have not yet pursued help.

### Statistical analysis

Firstly, a descriptive analysis was conducted to characterize the sample, using frequencies and percentages for categorical variables and means and standard deviations for continuous variables. Subsequently, Chi-square tests and Student's *t*-tests were used to evaluate gender differences. Secondly, Pearson correlation coefficients were calculated to analyze associations between the UCLA Loneliness Scale and the De Jong Gierveld Loneliness Scale (DJG) and the rest of the variables. Finally, a multiple linear regression analysis was performed to examine the relationship between DJG scale scores and the correlated variables, employing a stepwise method to refine the model and identify the best-fitting predictors (Núñez et al., 2011). This stepwise approach iteratively excluded the least correlated variables to optimize the statistical model (Hamilton, 1994). Data collection and analyses were carried out using Microsoft Excel and SPSS software (Version 29.0) (IBM Corp, 2023). Statistical significance was set at *p* < 0.05.

### Ethical aspects

This study was reviewed and approved by the Research Ethics Committee of the Autonomous Community of Aragon (CEICA; PI24/050) and authorized by the University of Zaragoza for data processing (RAT 2024-9). Online informed consent was obtained from all participants, integrated into the survey itself. For minors, consent was also obtained from their parents or guardians. The informed consent detailed the study's objectives and its anonymous nature, emphasizing that the results could not, under any circumstances, be linked to individual participants. No identifying information was collected, and the data were anonymized and processed in compliance with current personal data protection legislation. The risk/benefit analysis indicated no associated risks, as completing the survey posed no harm to participants. Additionally, no financial compensation was provided, as the data collected were used solely for the purpose of this project.

## Results

A total of 536 individuals participated in the survey, comprising 290 students (54.1%) from the University of Zaragoza and 246 students (45.9%) from Aragonese high schools (Table 1). The descriptive analysis revealed that 73.7% of respondents were women and 26.3% were men. The participants had a mean age of 20.09 years with a standard deviation of 3.67. For analytical clarity, ages were grouped into four categories: 14–17 years (24.6%), 18–19 years (23.5%), 20–22 years (28.9%), and 23–30 years (22.9%).

**Table 1:** Descriptive analysis of sociodemographic variables.

**Variables**		**Total (** ***n*** **: 536)**		**Female (** ***n*** **: 395)**		**Male (** ***n*** **: 141)**		***p***
		***n***	**%**	***n***	**%**	***n***	**%**	
Age	14–17	132	24.6	80	20.3	52	36.9	**< 0.001**
	18–19	126	23.5	94	23.8	32	22.7	
	20–22	155	28.9	122	30.9	33	23.4	
	23–30	123	22.9	99	25.1	24	17	
Current level of education	University	290	54.1	228	57.7	62	44	**0.005**
	High school	246	45.9	167	42.3	79	56	
Origin	Spain	519	96.8	383	97	136	96.5	0.768
	Foreigner	17	3.2	12	3	5	3.5	
Having a partner	Single	344	64.2	246	62.3	98	69.5	0.125
	In a relationship	192	35.8	149	37.7	43	30.5	
LGBTIQ+	No	412	76.9	300	75.9	112	79.4	0.536
	Yes	104	19.4	81	20.5	23	16.3	
	I'd rather not say it	20	3.7	14	3.5	6	4.3	
Living situation	Living with family/company	521	97.2	383	97	138	97.9	0.574
	Living alone	15	2.8	12	3	3	2.1	
Activities	Yes	200	37.3	125	31.6	75	53.2	**< 0.001**
	No	336	62.7	270	68.4	66	46.8	

LGBTIQ+: Lesbian, Gay, Bisexual, Transgender, Intersex, Queer/Questioning, and others.

Significant differences (p < 0.05) are highlighted in bold.

There were significant differences in age distribution by gender (*p* < 0.001), with a higher proportion of males in the youngest group (14–17 years: 36.9% vs. 20.3% in females) and a greater proportion of females in the 20–22 and 23–30 age groups. Regarding education level, university students were more likely to be female (57.7% vs. 44% in males, *p* = 0.005), whereas high school students had a higher proportion of males (56% vs. 42.3% in females).

In terms of relationship status, 64.2% of respondents identified as single, while 35.8% reported being in a romantic relationship. No significant gender differences were observed (*p* = 0.125). Regarding sexual orientation and identity, 19.4% identified as Lesbian, Gay, Bisexual, Transgender, Intersex, Queer/Questioning, and others (LGBTIQ+), while 76.9% identified as non-LGBTIQ+, and 3.7% preferred not to disclose this information. No significant differences were found between men and women (*p* = 0.536). The vast majority (97.2%) reported living with their families or in shared housing, with no significant gender difference (*p* = 0.574).

However, gender differences emerged in extracurricular activities (*p* < 0.001), where a higher percentage of men (53.2%) reported participating in associative, sporting, solidarity, or union activities compared to women (31.6%).

Regarding loneliness, 22.6% of respondents reported never feeling lonely, while 57.1% experienced loneliness sometimes, 18.7% frequently, and 1.7% always (Table 2). Gender differences were significant (*p* < 0.001), with men more likely to report never feeling lonely (34.8% vs. 18.2% in women), and women more frequently reporting occasional loneliness (63% vs. 40.4% in men). No significant gender differences were found in the self-reported duration of loneliness (*p* = 0.766), but more women (43.3%) than men (35.5%) reported experiencing loneliness for more than a year.

**Table 2:** Descriptive analysis of loneliness variables.

**Variables**		**Total (** ***n*** **: 536)**		**Female (** ***n*** **: 395)**		**Male (** ***n*** **: 141)**		***p***
		***n***	**%**	***n***	**%**	***n***	**%**	
Loneliness frequency	Never	121	22.6	72	18.2	49	34.8	**< 0.001**
	Sometimes	306	57.1	249	63	57	40.4	
	Frequently	100	18.7	68	17.2	32	22.7	
	Always	9	1.7	6	1.5	3	2.1	
Any stage of loneliness^a^	Never	16	3	8	2	8	5.7	0.406
	Almost never	69	12.9	39	9.9	30	21.3	
	Quite a lonely phase	30	5.6	20	5.1	10	7.1	
	Very lonely phase	6	1.1	5	1.3	1	0.7	
Loneliness duration	< 1 month	57	10.6	45	11.4	12	8.5	0.766
	1–3 months	57	10.6	47	11.9	10	7.1	
	3 months – 1 year	80	14.9	60	15.2	20	14.2	
	More than a year	221	41.2	171	43.3	50	35.5	
Help seeking	Yes	204	38.1	160	40.5	44	31.2	0.772
	No	211	39.4	163	41.3	48	34	
Age group loneliness	Teens (12–18)	171	31.9	133	33.7	38	27	0.896
	Young adults (19–30)	80	14.9	64	16.2	16	11.3	
	Mature adults (31–60)	10	1.9	7	1.8	3	2.1	
	Seniors (>60)	154	28.7	119	30.1	35	24.8	
Loneliness from COVID-19	Yes	113	21.1	91	23	22	15.6	0.712
	No	231	43.1	177	44.8	54	38.3	
	I don't know	71	13.2	55	13.9	16	11.3	
Social problem	Not important	1	0.2	1	0.3	0	0	0.161
	Slightly important	19	3.5	11	2.8	8	5.7	
	Quite important	194	36.2	155	39.2	39	27.7	
	Very important	201	37.5	156	39.5	45	31.9	
UCLA scale	Not lonely	248	46.3	195	49.4	53	37.6	0.634
	Lonely	167	31.2	128	32.4	39	27.7	
DJG scale	Not lonely	128	23.9	87	22	41	29.1	0.171
	Moderate loneliness	263	49.1	195	49.4	68	48.2	
	Severe loneliness	145	27.1	113	28.6	32	22.7	

^a^Answered by those who reported “Never” feeling alone.

UCLA Scale, University of California Los Angeles Loneliness Scale; DJG Scale, De Jong Gierveld Loneliness Scale.

Significant differences (p < 0.05) are highlighted in bold.

Regarding help-seeking for loneliness, 38.1% of participants had sought support, while 39.4% had not, with no significant gender differences (*p* = 0.772). When asked about the age group most associated with loneliness, 31.9% pointed to adolescence (12–18 years), followed by seniors over 60 (28.7%). No significant gender differences were observed in these perceptions (*p* = 0.896).

Perceived impact of the COVID-19 pandemic on loneliness was reported by 21.1% of participants, while 43.1% disagreed and 13.2% were unsure, with no gender-based differences (*p* = 0.712). When evaluating loneliness as a social issue, 37.5% of respondents considered it very important and 36.2% as quite important, again without significant gender differences (*p* = 0.161).

Loneliness scales also revealed gender variations. On the UCLA Loneliness Scale, men were more likely to be categorized as “not lonely” (37.6% vs. 49.4% in women), though this difference was not statistically significant (*p* = 0.634). The De Jong Gierveld Loneliness Scale (DJG) identified 23.9% of respondents as not lonely, 49.1% as experiencing moderate loneliness, and 27.1% as suffering from severe loneliness, with no statistically significant gender differences (*p* = 0.171).

The analysis of causes associated with loneliness revealed several contributing factors, with no significant gender differences in most variables (Table 3). A notable proportion of participants reported not having enough friends (16.8%), difficulty relating to others (25.2%), or not having time to socialize (21.6%). Females were more likely to cite lack of time to relate as a cause of loneliness (25.1% vs. 12.1%, *p* = 0.016). Other frequently reported causes included family conflicts (15.9%) and physical or mental health issues preventing social interactions (13.4%).

**Table 3:** Descriptive analysis of causes associated with loneliness.

**Variables**	**Total (** ***n*** **: 415** ^**a**^ **)**		**Female (** ***n*** **: 323)**		**Male (** ***n*** **: 92)**		***p***
	***n***	**%**	***n***	**%**	***n***	**%**	
Someone with whom I had a close relationship died, yes	30	5.6	22	5.6	8	5.7	0.624
I have changed my place of residence, yes	49	9.1	41	10.4	8	5.7	0.218
My family is in a bad financial situation, yes	10	1.9	10	2.5	0	0	0.077
My sexual orientation, yes	8	1.5	4	1	4	2.8	0.082
My physical/mental health prevents me from relating to others, yes	72	13.4	59	14.9	13	9.2	0.236
My disability situation, yes	8	1.5	5	1.3	3	2.1	0.348
I don't have a family, yes	2	0.4	2	0.5	0	0	0.431
I don't have enough friends, yes	90	16.8	73	18.5	17	12.1	0.404
I don't have time to relate, yes	116	21.6	99	25.1	17	12.1	**0.016**
I have difficulty relating to others, yes	135	25.2	103	26.1	32	22.7	0.544
I am an immigrant/I have migratory origins, yes	26	4.9	20	5.1	6	4.3	0.944
I suffer or have suffered bullying at school, yes	28	5.2	22	5.6	6	4.3	0.814
I suffer or have suffered cyberbullying, yes	8	1.5	6	1.5	2	1.4	0.900
I have conflicts with my family, yes	85	15.9	65	16.5	20	14.2	0.889
I have a bad financial situation, yes	9	1.7	5	1.3	4	2.8	0.132
I live alone, yes	19	3.5	13	3.3	6	4.3	0.389
Other, yes	23	4.3	19	4.8	4	2.8	0.444

^a^Answered by those who reported feeling alone.

Significant differences (p < 0.05) are highlighted in bold.

Variables related to social support, the use of social networks, self-esteem and self-perceived health were analyzed (Table 4). The analysis of social relationships revealed no significant gender differences in the perceived quantity and quality of family, friends, or classmates. Most participants reported having the desired amount of family (66.2%), friends (65.7%), and classmates (57.3%), with smaller proportions indicating “less than desired” relationships in these categories. Regarding quality, the majority rated their family relationships as good (71.3%), and only 6.5% perceived them as poor. Similarly, 97.8% reported good or fair quality in friendships.

**Table 4:** Descriptive analysis social support variables, use of social media and mental health.

**Variables**		**Total (** ***n*** **: 536)**		**Female (** ***n*** **: 395)**		**Male (** ***n*** **: 141)**		***p***
		***n***	**%**	***n***	**%**	***n***	**%**	
Family quantity	Less than desired	112	20.9	88	22.3	24	17.0	0.403
	As desired	355	66.2	258	65.3	97	68.8	
	More than desired	69	12.9	49	12.4	20	14.2	
Friends quantity	Less than desired	138	25.7	109	27.6	29	20.6	0.092
	As desired	352	65.7	257	65.1	95	67.4	
	More than desired	46	8.6	29	7.3	17	12.1	
Classmates quantity	Less than desired	165	30.8	128	32.4	37	26.2	0.375
	As desired	307	57.3	222	56.2	85	60.3	
	More than desired	64	11.9	45	11.4	19	13.5	
Family quality	Poor	35	6.5	27	6.8	8	5.7	0.830
	Fair	119	22.2	89	22.5	30	21.3	
	Good	382	71.3	279	70.6	103	73.0	
Friends quality	Poor	12	2.2	9	2.3	3	2.1	0.316
	Fair	111	20.7	88	22.3	23	16.3	
	Good	413	77.1	298	75.4	115	81.6	
Classmates quality	Poor	88	16.4	72	18.2	16	11.3	0.122
	Fair	203	37.9	150	38.0	53	37.6	
	Good	245	45.7	173	43.8	72	51.1	
Hours/day on mobile or social media	< 1 h	27	5.0	20	5.1	7	5.0	**0.033**
	1–3 h	237	44.2	160	40.5	77	54.6	
	3–6 h	218	40.7	173	43.8	45	31.9	
	>6 h	54	10.1	42	10.6	12	8.5	
Does social media abuse increase loneliness?	Yes	326	60.8	252	63.8	74	52.5	**0.043**
	No	61	11.4	44	11.1	17	12.1	
	Maybe	149	27.8	99	25.1	50	35.5	
Mental health issues	Yes	200	37.3	164	41.5	36	25.5	**< 0.001**
	No	336	62.7	231	58.5	105	74.5	
Have you sought help?	Yes	114	21.3	96	24.3	18	12.8	0.349
	No	86	16.0	68	17.2	18	12.8	

Significant differences (p < 0.05) are highlighted in bold.

Regarding hours spent on mobile or social media, females were more likely to use social media for 3–6 h daily (43.8% vs. 31.9%), while males were more likely to use it for 1–3 h daily (54.6% vs. 40.5%; *p* = 0.033). A higher percentage of females (63.8%) than males (52.5%) believed that excessive social media use increased loneliness (*p* = 0.043).

A higher percentage of females (41.5%) than males (25.5%) reported having mental health issues (*p* < 0.001), though no significant difference was found in seeking professional help (24.3% females vs. 12.8% males; *p* = 0.349).

Moreover, the descriptive analysis of the quantitative variables was carried out, the mean and standard deviation were analyzed (Table 5). The mean age was 20 years, the mean score on the UCLA Loneliness Scale was 5 (corresponding to “not lonely”), and the mean score on the DJG scale was 2 (“moderate loneliness”). An average of 2 causes associated with the feeling of loneliness was found. Regarding gender differences, males were slightly younger on average than females (19.48 vs. 20.30 years, *p* = 0.022). While no gender differences were observed in the number of loneliness causes or UCLA Loneliness Scale scores, males scored lower on the DJG compared to females (2.77 vs. 3.18, *p* = 0.026). Males also reported significantly higher self-esteem (3.33 vs. 2.98, *p* = 0.001) and better self-perceived health (4.01 vs. 3.82, *p* = 0.010).

**Table 5:** Descriptive analysis of quantitative variables.

**Variables**	**Total**		**Female**		**Male**		***p***
	***M***	**SD**	***M***	**SD**	***M***	**SD**	
Age	20.09	3.670	20.30	3.473	19.48	4.126	**0.022**
Number of causes of loneliness	2.01	1.281	2.02	1.321	2.00	1.150	0.916
UCLA scale	5.04	1.550	5.00	1.533	5.18	1.610	0.322
DJG scale	3.07	1.868	3.18	1.836	2.77	1.929	**0.026**
Self-esteem	3.07	1.099	2.98	1.093	3.33	1.079	**0.001**
Health	3.87	0.771	3.82	0.754	4.01	0.802	**0.010**

UCLA Scale, University of California Los Angeles Loneliness Scale; DJG Scale, De Jong Gierveld Loneliness Scale.

Significant differences (p < 0.05) are highlighted in bold.

Next, the correlation analysis of the UCLA Loneliness Scale and the De Jong Gierveld Loneliness Scale with the other variables was performed (Table 6). A higher level of loneliness according to the UCLA Loneliness Scale is positively correlated with belonging to the LGBTIQ+ community (*r* = 0.156, *p* < 0.001), being a high school student (*r* = 0.137, *p* < 0.05), loneliness frequency (*r* = 0.569, *p* < 0.001), loneliness duration (*r* = 0.361, *p* < 0.001), and the total number of causes of loneliness (*r* = 0.420, *p* < 0.001), friend quantity (*r* = −0.316, *p* < 0.001) and quality (*r* = −0.288, *p* < 0.001), family quality (*r* = −0.145, *p* < 0.01), classmates quantity (*r* = −0.176, *p* < 0.001), and quality (*r* = −0.203, *p* < 0.001), self-esteem (*r* = −0.328, *p* < 0.001), health (*r* = −0.335, *p* < 0.001), and having a mental health problem (*r* = 0.303, *p* < 0.001).

**Table 6:** Correlation of the variables with the UCLA Loneliness Scale and the De Jong Gierveld Loneliness Scale.

**Variables**	**UCLA total**	**DJG total**
UCLA total	1	0.659^**^
JG total	0.659^**^	1
Age	−0.074	−0.045
Age category	−0.119^*^	−0.095
Gender	0.072	−0.056
Municipality	0.100	0.046
Origin	0.059	−0.001
Having a partner	−0.037	−0.053
LGBTIQ+	0.156^**^	0.164^**^
Current level of education	0.137^*^	0.071
Living situation	0.007	0.019
Activities	−0.009	0.096
Loneliness frequency	0.569^**^	0.537^**^
Any stage of loneliness^a^	–	0.323^**^
Loneliness duration	0.361^**^	0.335^**^
Help seeking	0.037	0.096
Cause sum	0.420^**^	0.358^**^
Family quantity	−0.004	−0.059
Friend quantity	−0.316^**^	−0.422^**^
Classmates quantity	−0.176^**^	−0.096
Family quality	−0.145^**^	−0.236^**^
Friend quality	−0.288^**^	−0.344^**^
Classmates quality	−0.203^**^	−0.231^**^
Social media hours	0.088	0.113^*^
Self-esteem	−0.328^**^	−0.399^**^
Health	−0.335^**^	−0.397^**^
Mental health	0.303^**^	0.357^**^
Mental health help	0.025	−0.072

LGBTIQ+, Lesbian, Gay, Bisexual, Transgender, Intersex, Queer/Questioning, and others. UCLA, University of California Los Angeles Loneliness Scale; DJG, the De Jong Gierveld Loneliness Scale.

^a^Answered by those who reported “Never” feeling alone.

The ^*^ indicates a significant correlation (p < 0.05), while ^**^ indicates a highly significant correlation (p < 0.001).

For the De Jong Gierveld Loneliness Scale, a higher level of loneliness is positively correlated with belonging to the LGBTIQ+ community (*r* = 0.164, *p* < 0.001), loneliness frequency (*r* = 0.537, *p* < 0.001), loneliness duration (*r* = 0.335, *p* < 0.001), and the total number of causes of loneliness (*r* = 0.358, *p* < 0.001). It is also negatively correlated with the quantity (*r* = −0.422, *p* < 0.001) and quality (*r* = −0.344, *p* < 0.001) of friends, family quality (*r* = −0.236, *p* < 0.001), classmates quality (*r* = −0.231, *p* < 0.001), hours spend on social media (*r* = 0.113, *p* < 0.05), self-esteem (*r* = −0.399, *p* < 0.001), health (*r* = −0.397, *p* < 0.001), and mental health (*r* = 0.357, *p* < 0.001).

Finally, the regression analysis identified key predictors of loneliness, as measured by the De Jong Gierveld Loneliness Scale (Table 7). Better self-perceived health [*B* = −0.416, β = −0.173, *p* < 0.001, 95% CI (−0.626, −0.205)], a greater number of friends [*B* = −0.809, β = −0.246, *p* < 0.001, 95% CI (−1.061, −0.558)], higher-quality friendships [*B* = −0.594, β = −0.156, *p* < 0.001, 95% CI (−0.883, −0.305)], and higher self-esteem [*B* = −0.225, β = −0.134, *p* = 0.002, 95% CI (−0.367, −0.083)] were significantly associated with lower levels of loneliness. In contrast, more mental health problems [*B* = 0.516, β = 0.134, *p* = 0.002, 95% CI (0.188, 0.843)] and more hours spent on social media [*B* = 0.207, β = 0.083, *p* = 0.029, 95% CI (0.021, 0.392)] were associated with higher loneliness scores. The regression model explained 28.7% of the variance in loneliness (adjusted *R*^2^ = 0.287, F_(6, 509)_ = 35.561, *p* < 0.001).

**Table 7:** Linear regression analysis of variables associated with the De Jong Gierveld Loneliness Scale.

**Variables**	**Unstandardized coefficients**		**Standardized coefficients**	***t***	**Sig**.	**95.0% confidence interval for B**		**Collinearity statistics**	
	***B***	**Std. error**	**Beta**			**Lower bound**	**Upper bound**	**Tolerancia**	**VIF**
(Constant)	7.748	0.628		12.347	**< 0.001**	6.515	8.981		
Health	−0.416	0.107	−0.173	−3.877	**< 0.001**	−0.626	−0.205	0.697	1.435
Friend quantity	−0.809	0.128	−0.246	−6.319	**< 0.001**	−1.061	−0.558	0.915	1.093
Mental health	0.516	0.167	0.134	3.089	**0.002**	0.188	0.843	0.738	1.355
Friend quality	−0.594	0.147	−0.156	−4.041	**< 0.001**	−0.883	−0.305	0.933	1.072
Self-esteem	−0.225	0.072	−0.134	−3.121	**0.002**	−0.367	−0.083	0.754	1.326
Social media hours	0.207	0.095	0.083	2.186	**0.029**	0.021	0.392	0.967	1.034

Dependent variable, DJG, De Jong Gierveld Loneliness Scale. Significant differences (p < 0.05) are highlighted in bold.

## Discussion

In this study, we examined feelings of loneliness among 536 adolescents and young adults in our community. Of the participants, 20.4% reported feeling lonely frequently or always. Loneliness was found to be associated with time spent on social media, the quantity and quality of friendships, low self-esteem, and poorer physical and mental health. When dividing the sample by gender, the majority of participants were women. This may be attributed to the higher likelihood of women participating in surveys compared to men (Smith, 2008). Regarding age, participants ranged from 14 to 30 years old, with 54.6% falling within the typical age range of Spanish university students (18–21 years), consistent with this sample (Ministerio de Universidades, 2023).

Significant gender differences were observed in various variables, including age and level of education (with most boys in high school and most girls in university), extracurricular activities (boys participated in more activities), frequency of loneliness (a higher proportion of men reported never feeling lonely), hours spent on social media (higher in women), self-esteem (higher in men), perception of health status (better in men), and mental health problems (more frequent in women). Additionally, women scored higher on the De Jong Gierveld Loneliness Scale (DJG) when treated as a continuous measure. However, no significant correlation was found between gender and loneliness levels on either the University of California Los Angeles Loneliness Scale (UCLA Loneliness Scale) or the DJG Scale. Given the relationship between these characteristics and loneliness, one might expect women to have higher loneliness scores than men. However, our findings align with some studies (Maes et al., 2019) while differing from others that report higher loneliness prevalence among women (Tuñón et al., 2023).

More than half of young people reported feeling lonely sometimes, and one in five frequently or always. Approximately 41.2% had experienced loneliness for more than a year, underscoring the need for effective interventions to address this issue. Additionally, nearly all participants considered loneliness to be a quite or very important social problem. Correlation and regression analyses revealed that higher frequency and longer duration of loneliness were associated with higher scores on the loneliness scales, indicating greater loneliness levels. At least one in five young people believed their loneliness was exacerbated or caused by the COVID-19 pandemic, a period marked by increased isolation and a rise in mental health issues (Wilkialis et al., 2021). The general consensus among participants was that loneliness is most prevalent among adolescents and individuals over 60 years of age. Only 1.9% associated significant loneliness with adults aged 31 to 60 years. These findings align with previous studies [State Observatory of Unwanted Loneliness (SoledadES), 2022] and reflect the “U-shaped” distribution of loneliness across the lifespan (Martín Roncero and González-Rábago, 2021). Despite recognizing loneliness as a serious issue, about half of the participants had not sought help. This discrepancy may stem from a lack of perceived or actual close relationships to rely on, a lack of tools or resources for seeking support, or the stigma surrounding loneliness, which might make individuals hesitant to discuss it even with family members.

The UCLA Loneliness Scale classified 46.3% of participants as non-lonely and 31.2% as lonely, while the De Jong Gierveld Loneliness Scale identified 49.1% as experiencing moderate loneliness, 27.1% as severe loneliness, and only 23.9% as non-lonely. These findings are similar to those of the Red Cross of Catalonia's study, where only 20.25% of young people aged 18 to 29 were categorized as non-lonely according to the De Jong Gierveld Loneliness Scale (Catalonia Red Cross, 2022). However, they differ significantly from other studies reporting a lower prevalence of youth loneliness, with rates of 25.5% or less (Casal-Rodríguez et al., 2023; Tuñón et al., 2023). The study also examined numerous causes that participants linked to their feelings of loneliness. The most common were “I have difficulty relating to others,” “I don't have time to socialize,” “I don't have enough friends,” and “I have conflicts with my family.” These associations align with prior research findings (Casal-Rodríguez et al., 2023; Tuñón et al., 2023). While there is substantial knowledge about the protective factors and risk factors for loneliness and social isolation in older adults, these may not fully apply to younger populations. Further research is needed to identify the specific factors most relevant to loneliness in young people.

Regarding mobile phone and social network use, approximately half of the young people in the study reported spending at least 3 h daily on these platforms, with one in 10 spending 6 h or more. These findings differ significantly from the 2023 Annual Social Media Study, which reported that adolescents spend an average of 1 h and 14 min, and young adults 1 h and 32 min (IAB Spain, 2023). Correlation analysis revealed that increased time spent on mobile phones and social networks was associated with higher levels of loneliness, consistent with other studies (Pérez and Quiroga-Garza, 2019; Sota-Velásquez et al., 2021). This may be explained by reduced time available for face-to-face social interactions. Additionally, most participants (60.8%) believed that excessive internet use could promote loneliness. Given that younger individuals are gaining earlier access to technology, it is crucial to address this as a potential risk factor for youth loneliness and to promote healthy usage patterns to protect social relationships. Lower levels of self-esteem were also found to correlate with greater loneliness. A lack of confidence may hinder socialization, while negative self-perceptions could lead individuals to misinterpret their relationships as inadequate or insufficient. Similarly, those with poorer self-perceived health and mental health problems reported significantly higher levels of loneliness. Participants often attributed their loneliness directly to health issues, consistent with previous findings (Martín Roncero and González-Rábago, 2021; Tuñón et al., 2023). Poor health may limit social interactions, fostering feelings of loneliness, while loneliness itself has well-documented negative effects on health (Martins Barroso et al., 2023). Interestingly, no significant correlation was found between loneliness and relationship status, contrasting with prior studies where having a partner served as a protective factor against loneliness (Hernan-Montalban and Rodríguez-Moreno, 2017; Luhmann and Hawkley, 2016). However, belonging to the LGBTIQ+ community emerged as a significant risk factor for loneliness, with participants in this group scoring higher on both the UCLA Loneliness Scale and the De Jong Gierveld Loneliness Scale. This association is likely influenced by the discrimination and social challenges faced by this minority group. Overall, loneliness is increasingly recognized as a risk factor for various diseases (Mann et al., 2022; Matthews et al., 2017).

Given the high prevalence and duration of loneliness among adolescents and young adults observed in this study, practical steps are needed to address the issue systematically. First, integrating loneliness screenings into school and university health programs could help in early identification of at-risk individuals. Second, the development of targeted social media literacy curricula may mitigate the negative impact of excessive online activity on social connection. Third, promoting structured opportunities for social engagement, particularly for vulnerable groups such as LGBTIQ+ youth, can serve as protective buffers. Finally, training school staff and mental health professionals to recognize and address loneliness as a legitimate concern could improve both detection and support outcomes.

### Limitations

This study analyzed a total of 536 surveys, exceeding the calculated sample size of 292 needed to achieve statistical significance. This larger sample size enhances the external validity of the project, allowing for more robust inference of the results. However, the use of non-probabilistic convenience sampling meant that participants voluntarily chose to respond to the survey. This approach could potentially affect the representativeness of the sample and, consequently, the external validity of the study. Furthermore, it may have introduced self-selection bias, as individuals who feel more connected to the topic of loneliness may have been more inclined to participate, potentially inflating the prevalence of loneliness in the sample.

The study utilized an online form, incorporating questions and questionnaires that have been validated in similar contexts. One of the scales used, the De Jong Gierveld Loneliness Scale, has been validated in Spain for use with elderly populations but not for young people. Due to the lack of a validated loneliness scale tailored to youth, the De Jong Gierveld Loneliness Scale was chosen. This limitation underscores the need for instruments specifically designed to measure loneliness in younger populations.

Additionally, as the data were obtained through self-report measures, the results may be subject to social desirability and recall biases. Participants might have under- or over-reported certain behaviors or experiences, either unintentionally or in an effort to present themselves in a more socially acceptable manner. Finally, since the study was conducted in Zaragoza, Spain, the generalizability of the findings to other regions or countries may be limited due to cultural, social, or contextual differences. Further studies in diverse settings would be necessary to confirm the broader applicability of the results.

## Conclusions

Loneliness is highly prevalent among young students in Aragón, affecting two-thirds of individuals aged 14 to 30, with at least half experiencing loneliness for over a year. Higher loneliness levels are associated with spending more time on social media, fewer and lower-quality relationships, lower self-esteem, poorer self-perceived health, and having mental health problems. While no significant gender or age differences were found, the UCLA Loneliness Scale identified 31.2% of participants as lonely, and the De Jong Gierveld Scale classified 49.1% with moderate loneliness and 27.1% with severe loneliness. The lack of a youth-specific loneliness scale highlights the need for tailored tools to better detect, prevent, and address loneliness in this age group through social, health, and educational services.

## Data Availability

The raw data supporting the conclusions of this article will be made available by the authors, without undue reservation.
